# Potential Roles of Adiponectin Isoforms in Human Obesity with Delayed Wound Healing

**DOI:** 10.3390/cells8101134

**Published:** 2019-09-24

**Authors:** Jiyoon Ryu, Colleen A. Loza, Huan Xu, Min Zhou, Jason T. Hadley, Jielei Wu, Huayu You, Huaiqing Wang, Jihong Yang, Juli Bai, Feng Liu, Christie Bialowas, Lily Q. Dong

**Affiliations:** 1 Department of Cell Systems & Anatomy, University of Texas Health Science Center, San Antonio, TX 78229-3900, USA; 2 Department of Pharmacology, University of Texas Health Science Center, San Antonio, TX 78229-3900, USA; 3 Division of Plastic Surgery, Department of Surgery, Albany Medical Center, 50 New Scotland Ave, 1st Floor, Albany, NY 12208-3403, USA

**Keywords:** human, woman, adiponectin, wound healing, multimers

## Abstract

Adiponectin is an adipokine with anti-insulin resistance and anti-inflammatory functions. It exists in serum predominantly in three multimeric complexes: the trimer, hexamer, and high-molecular-weight forms. Although recent studies indicate that adiponectin promotes wound healing in rodents, its role in the wound healing process in humans is unknown. This study investigated the expression levels of adiponectin in adipose tissue and serum of women who experienced either normal or delayed wound healing after abdominal plastic surgery. We found that obese women with delayed healing had slightly lower total adiponectin levels in their adipose tissue compared with women with normal healing rates. Among the different isoforms of adiponectin, levels of the trimer forms were significantly reduced in adipose tissue, but not the serum, of obese women with delayed healing compared to women who healed normally. This study provides clinical evidence for a potential role of low-molecular-weight oligomers of adiponectin in the wound healing process as well as implications for an autocrine and/or paracrine mechanism of adiponectin action in adipose tissues.

## 1. Introduction

Obesity, characterized by excess body fat, has become a significant public health problem worldwide. Defined as a body-mass index (BMI) of at least 30 kg/m^2^, obesity is a chronic illness that can produce detrimental effects on multiple organ systems of the body. As the prevalence of obesity increases, so does the burden of its associated comorbidities [1]. A major complication of obesity is impaired wound healing [2]. Clinical records indicate that the wound healing process for some obese patients is extremely slow, contributing to a higher risk of infection and diminished quality of life among affected patients [3]. No treatment currently exists to help to promote wound healing in obese patients after surgery.

Wound healing is a complex process that involves a cascade of precisely timed and coordinate events that regulate cell interactions and facilitate tissue repair. These time-dependent steps are separated into four phases: (1) coagulation and hemostasis, which serves to stop bleeding at the site of injury and protect the vasculature; (2) the inflammatory phase, which occurs shortly after injury and functions to clear the injury site of debris and pathogens; (3) the proliferative phase, which lasts for approximately two weeks following injury and functions to prepare the injury for tissue repair; and (4) the remodeling phase, which occurs weeks to years following injury and serves to restore tissue integrity and function [4]. Wound healing takes place in all three layers of the skin: the epidermis, the dermis, and the subcutaneous adipose tissue. Although once thought to be an inert, energy-storage depot, adipose tissue is now recognized as a major endocrine organ in the body, secreting a plethora of bioactive factors known as adipokines [5]. Several studies suggest that adipose tissue is an active participant in wound repair [6,7,8], though the specific role of adipocytes in the full process is not well understood. Adipose tissue extracts, when seeded over skin wounds, have been demonstrated to promote of wound repair [9]. Indeed, adipose tissue likely participates in wound healing through paracrine signaling, implicating the important role of secreted factors (adipokines) in the wound healing process [10].

Human adiponectin is a small protein containing 244 amino acids that is secreted selectively from adipocytes and that has anti-inflammatory and insulin-resistance properties [11,12]. Adiponectin exists in adipose tissues in four isoforms: the monomer (29 kDa), trimer (67 kDa), hexamer (130 kDa), and high-molecular-weight (HMW) oligomer (300 kDa) [12]. The monomer is the structural base for further assembly into oligomers of various molecular weights [13]. Functionally, each isoform has distinct biochemical and biological characteristics [13,14,15,16]. The HMW oligomer has been associated with anti-inflammatory, insulin-sensitizing, and antidiabetic properties in humans [12,17,18]. Acting in peripheral tissues and the central nervous system, the hexamer and trimer forms of adiponectin can regulate lipid metabolism, influence energy expenditure, and produce antidepressant-like effects [19,20]. Weight loss in severely obese individuals produces overall increases in total and the monomeric forms of adiponectin [21]. Serum levels of adiponectin are decreased in individuals with obesity and obesity-related diseases [22]. Indeed, adiponectin can impact various physiological processes via the unique roles played by each adiponectin isoform.

Patients with diabetic foot ulcers have shown lower plasma levels of adiponectin [23]. Consistent with this view, animal studies have demonstrated that wound closure is significantly delayed in adiponectin deficient mice compared with wild-type mice [24]. Recent studies also indicate that adiponectin can promote wound repair in human bronchial epithelial cell lines [25] and wound healing in the cornea [26]. Administration of adiponectin, either systemically or topically, has ameliorated impaired wound healing in adiponectin knockout mice and diabetic db/db mice [24]. However, the correlation between adiponectin expression and wound healing in humans is unknown, as is which isoform of adiponectin has potential effects in mediating this relationship.

In the current study, we examined the tissue and serum levels of total adiponectin as well as each of its isoforms in normal-weight and obese women with normal or delayed wound healing. We found that the levels of the trimer form of adiponectin were significantly reduced in adipose tissues of obese patients with delayed wound healing compared to those of non-obese and obese patient with normal wound healing rate. Our study reveals a potential role of this adiponectin isoform in promoting the wound healing process in humans.

## 2. Materials and Methods

### 2.1. Patients

Patients undergoing abdominal plastic surgery at our facility were eligible for inclusion. The Institutional Review Board of the University of Texas Health Science Center approved the study protocol (#HSC20160323N), and all patients provided written informed consent for inclusion.

### 2.2. Human Tissue Collection

We collected abdominal adipose tissue and blood samples from all included patients. All tissues were washed with cold 1xPBS after collection. Skin was detached from surrounding adipose tissues. Using sterile surgical tools, subcutaneous adipose tissues were cut and separated into 300 mg portions, devoid of blood vessels and connective tissue, and were immediately submerged into liquid nitrogen. Tissues were stored for the long term at −80 °C. Blood from each patient was centrifuged at 3500 rpm for 15 min. Serum was then collected, aliquoted, and stored at −80 °C.

Clinical data collected included a numeric patient identifier, BMI, blood glucose level, hemoglobin A1c concentration, diabetes status, cancer status, age, sex, surgery date, and time to subsequent wound healing. For BMI, 18.5–24.9 kg/m^2^ was considered normal, and >30 kg/m^2^ was considered obese. Wounds were considered healed when all three layers of the abdominal skin had closed completely. The clinical timeframe for normal wound healing was defined as 20–45 days, and 90+ days for delayed wound healing [27].

### 2.3. RNA Isolation and Real-Time Polymerase Chain Reaction Analysis

Total RNA was isolated from human adipose tissues using TRIzol reagent (Invitrogen, Waltham, MA, USA) in accordance with the manufacturer’s instructions. RNA concentration and quality were detected using a NanoDrop 2000 spectrophotometer (Thermo Fisher Scientific, Waltham, MA, USA). RNA isolated from TRIzol reagent was used to generate cDNA via Qiagen QuantiTect Reverse Transcription Kit (Qiagen, Hilden, Germany). cDNA was diluted to a 1:10 concentration and quantified by NanoDrop 2000 spectrophotometry to ensure equivalent cDNA concentrations among samples. The forward primer (5′ TGACCAGGAAACCACGACTC 3′) and reverse primer sequences (5′ CTCCGGTTTCACCATGTCT 3′) were generated for adiponectin using the Primer-Blast tool (National Center for Biotechnology Information (NCBI); https://www.ncbi.nlm.nih.gov/tools/primer-blast/), and 100% human adiponectin RNA sequence identity match was confirmed using NCBI nucleotide blast. The forward primer sequence 5′ GTCATTCCAAATATGAGATGCGT 3′ and reverse primer sequence 5′ GCTATCACCTCCCCTGTGTG 3′ of actin were used. cDNA from the obese patients with normal and delayed wound healing (see clinical data collected, above) was analyzed by the CFX96 Touch Real-Time Polymerase Chain Reaction Detection System (RT-PCR; Bio-Rad Laboratories, Inc., Hercules, CA, USA) using adiponectin primers and actin primers (as an endogenous control) (Sigma-Aldrich, St. Louis, MO, USA).

### 2.4. Western Blot Analysis

Adipose tissue lysates were prepared by adding approximately 300 mg of abdominal adipose tissue to 2000 µL of homogenization buffer and homogenizing on ice until the tissue was completely immersed. After homogenization, the tissue samples were incubated on ice for 30 min. The samples were then spun down at 14,000× *g* for 10 min at 4 °C. The lipid layers in the lysates were removed from the top of the samples, and the protein lysates were collected in a new tube. The protein concentration of the tissue lysates was quantified by Bradford assay. For preparation of reducing samples, tissue lysates were diluted to 1.8 μg/µL with a PBS (Phosphate Buffered Saline) and SDS (Sodium Dodecyl Sulfate) sample buffer in the presence of β-mercaptoethanol, and serum samples were diluted 50 times with the same buffer. The samples were heated at 95 °C for 10 min, and total adiponectin levels in the adipose tissues and sera were detected using SDS-PAGE and Western blotting using anti-adiponectin antibodies (R&D systems; Cat. NO. MAB10652). To measure the levels of adiponectin isoforms (monomer, dimer, trimer, hexamer, and HMW forms) in adipose tissues and sera, we prepared the samples using a nonreducing method, in which β-mercaptoethanol was excluded from the SDS sample buffer. The samples were either denatured by heating at 95 °C for 10 min or maintained as nondenatured with no heating. Adiponectin levels were detected on mini Protein TGX precast gels (4%–15%, Bio-Rad Laboratories, Inc. Hercules, CA, USA) and Western blot analysis. In brief, the entire gradient gel was transferred to a nitrocellulose membrane at 400 mA for 1 h. Sample loading was normalized with β-actin (Cell signaling; Cat. NO. 4970) control for tissue samples and albumin control for serum samples.

### 2.5. Data Analysis

ImageJ was used to quantify Western blot band intensities. Results from RT-PCR were calculated using the delta-delta Ct method. Data are represented as the group mean ± SEM. *p* values were calculated using one-way ANOVA employing the GraphPad Prism 7.04 software. A *p* value of < 0.05 was considered statistically significant.

## 3. Results

### 3.1. Patients

A total of 12 women underwent abdominal plastic surgery at our facility. Since there were no available non-obese wound healing delay rate patients, we divided them into three groups based on their BMI and times to postsurgical wound healing: non-obese patients with a normal wound healing rate (Non-Obese–Normal, n = 3); obese patients with a normal wound healing rate (Obese–Normal; n = 5); and obese patients with delayed wound healing (Obese–Delayed; n = 4) (Table 1). Among these 12 patients, 6 of them had Type 2 diabetes (3 each in the Obese–Normal and Obese–Delayed groups). Additionally, one patient in the Obese–Delayed group had a history of cancer.

### 3.2. Wound Healing Timeframes

**Figure 1:**
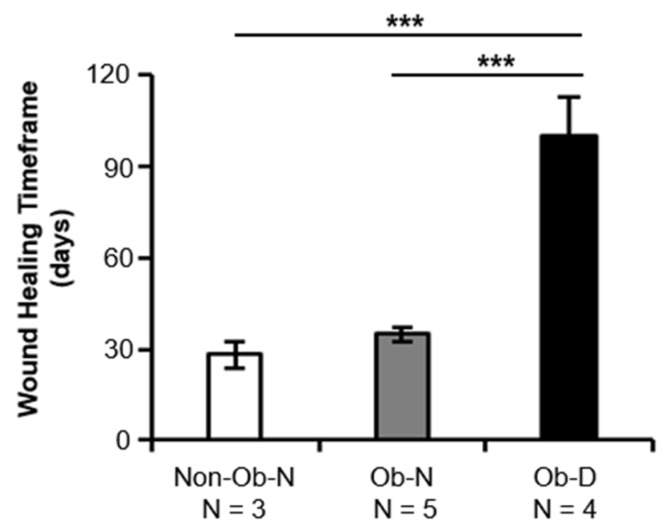
Wound healing timeframes for non-obese patients with a normal wound healing rate (Non-Ob–N; n = 3), obese patients with a normal wound healing rate (Ob–N; n = 5), and obese patients with delayed wound healing (Ob–D; n = 4). The clinical timeframe for normal wound healing was defined as 20–45 days, and for delayed wound healing, 90+ days. *** *p* < 0.001.

Wound healing timeframes differed significantly between the obese women with delayed healing and the other two groups (*p* < 0.001 vs. the Obese–Normal patients and *p* < 0.001 vs. the Non-Obese–Normal patients; Figure 1). No significant difference in wound healing timeframes was observed between the Non-Obese–Normal and Obese–Normal patients (*p* = 0.83), suggesting that some obese patients heal within a time period more similar to that of normal-weight patients, while some experience severely impaired wound healing.

### 3.3. Levels of Total Adiponectin Expression

**Figure 2:**
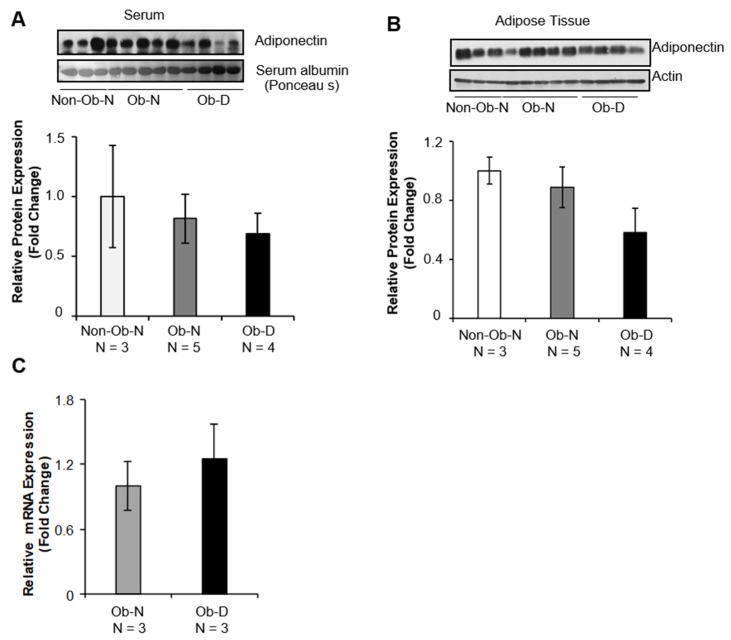
Total adiponectin protein and mRNA levels in non-obese and obese patients. Total adiponectin levels were detected in (**A**) sera and (**B**) adipose tissue from Non-Obese–Normal (Non-Ob–N; n = 3), Obese–Normal (Ob–N; n = 5), and Obese–Delayed (Ob–D; n = 4) patients using SDS-PAGE and Western blotting. Albumin was used as the loading control for serum, and actin was used as the loading control for adipose tissues. (**C**) mRNA levels of adiponectin in adipose tissue from three of the Obese–Normal (Ob–N) and three of the Obese–Delayed (Ob–D) patients.

Although total serum adiponectin levels were reduced in obese patients, regardless of wound healing rate, there were no significant differences among the three groups of patients (Figure 2A). The total adiponectin level in adipose tissue was also slightly lower in obese patients with delayed healing than in either the Non-Obese–Normal or Obese–Normal patients (Figure 2B). Given that tissue adiponectin mRNA levels were comparable between the Obese–Normal group and the Obese–Delayed group (Figure 2C), the differences in adiponectin expression appear to reflect a post-transcriptional and/or post-translational mechanism.

### 3.4. Levels of Adiponectin Isoform Expression

**Figure 3:**
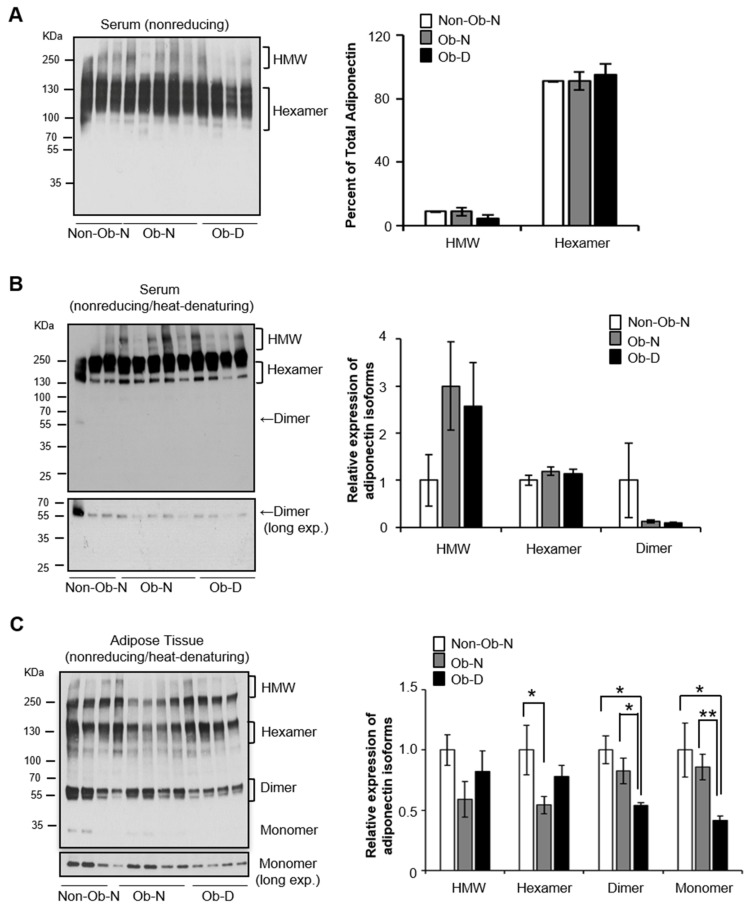
Adiponectin isoform levels. (**A**) Representative image and graphical presentation of adiponectin isomers detected in serum by nonreducing gradient SDS-PAGE and Western blotting. Representative image and graphical presentation of adiponectin isomers detected in (**B**) serum and (**C**) adipose tissue by nonreduced and heat-denatured gradient SDS-PAGE and Western blotting (* *p* < 0.05 and ** *p* < 0.01).

Under the nonreduced and non-denatured condition, serum levels of the hexamer and HMW forms of adiponectin were similar among the three groups (Figure 3A), consistent with the total serum adiponectin levels (Figure 2A). The trimer form of adiponectin was not detectable in the serum of any group under this condition (Figure 3A) due to the limited amount of the trimer form relative to the HMW form in the circulatory system of women [16]. Interestingly, we observed the dimer form, but not trimer or monomer forms, in human serum when the samples were prepared under nonreduced and heat-denatured conditions (Figure 3B). These data are consistent with cellular studies indicating that HMW and hexamer forms of adiponectin composed of disulfide-linked dimers.

We then examined the levels of adiponectin isoforms in adipose tissue under nonreduced and heat-denatured conditions. In contrast to serum samples, we detected both the dimer and monomer isoforms in adipose tissue (Figure 3C). Trimeric adiponectin can dissociate into 2:1 dimer to monomer ratio in vitro under nonreduced and heat-denatured conditions [15]. Levels of both the dimer and monomer forms of adiponectin were significantly decreased in the adipose tissue of the obese patients with delayed wound healing compared to the other two groups (Figure 3C), suggesting that higher tissue levels of the trimer isoform, rather than the HMW or hexamer form, of adiponectin are associated with faster wound healing. Additionally, tissue levels of the HMW and hexamer adiponectin forms were reduced in the obese group with normal healing compared with the normal-weight patients (Figure 3C), suggesting that alterations in these forms more closely relate to obesity than to the wound healing process.

## 4. Discussion

Adipose tissue is an endocrine organ that secretes various types of adipokines that control energy homeostasis and inflammation locally as well as systemically. Adiponectin is an important adipokine with anti-insulin resistance and anti-inflammatory functions [11,12,28]. In addition, adiponectin has been shown to play a role in accelerating cutaneous wound healing in mice via systemic injection and topical treatment of adiponectin [24,29] and enhancing re-epithelization of injured corneal via adiponectin eyedrops [26]. At the cellular level, adiponectin treatment was sufficient to induce proliferation and migration in wound repair-related cell types including keratinocytes [24] and corneal epithelial cells [26]. The effects of adiponectin on wound healing are mediated via AdipoR1/AdipoR2 receptors and the ERK signaling pathway [24,29]. These data suggested that adiponectin might act as a stimulatory factor in the proliferation and migration process during wound healing.

While accumulating data suggest that adiponectin promotes wound healing in rodents [24,26], the relation of adiponectin levels, and specifically its isoform levels, to wound healing in humans remains unclear. In the present study, we report that the trimeric form of adiponectin was significantly decreased in the abdominal adipose tissues of obese women with delayed wound healing compared to women with normal-body weight or women who are obese women but had normal healing rates. The reduction in the trimer form of adiponectin was detected only in adipose tissue, but not in the serum, of women with delayed wound healing.

Adiponectin exists in circulation as three different forms—trimer, hexamer, and high-molecular-weight (HMW)—each of which has distinct biological functions. HMW form is correlated with enhanced insulin sensitivity and anti-inflammation [12,19,20,30]. Administration of the HMW and hexamer forms, but not the trimer form, is sufficient to activate NF-κB in C2C12 myoblast cells [15], while the trimer and globular adiponectin (gAd) forms enhance AMPK activation in skeletal muscle tissue and myocytes [15,31]. It has been reported that the smallest adiponectin oligomer under native condition is the trimer [13,32,33] and that trimer is formed by the globular domain of human and mouse adiponectin [34,35]. In the current study, we found that levels of trimeric adiponectin were selectively reduced in abdominal adipose tissue of obese women who had significantly delayed wound healing compared with women who had normal-body weight or those who were obese but with normal healing rates. Consistent with our finding, administration of the globular form of adiponectin, which is likely to form trimers [36], was found to be sufficient to promote wound healing at cellular levels as well as in rodent models by enhancing proliferation and migration in keratinocytes and epithelial cells [24,25,26]. Our data reveal a positive correlation between trimer expression in adipose tissues and the speed of wound healing in humans, suggesting a potential autocrine and/or paracrine role of trimer in regulating adipose function.

Cellular and in vitro studies indicate that trimeric adiponectin can be dissociated into dimer and monomer forms under nonreduced SDS-PAGE conditions, and that the HMW and hexamer forms can collapse only into the dimer form [15]. Consistent with these findings, we detected both dissociated monomeric and dimeric adiponectin in the adipose tissues, but only dimeric adiponectin in the serum, under nonreduced conditions. These findings suggest that (1) the trimer form is the source of dimeric and monomeric adiponectin in adipose tissues and (2) the HMW and hexamer forms are the source of dimeric adiponectin in serum. These findings have several implications. First, the population of trimer adiponectin comparted to HMW and hexamer in serum was much less than that comparted in adipose tissue in our study. Since all forms of adiponectin can be secreted from cultured adipocytes [19,37,38,39], the trimer isoform may perform its function locally in an autocrine and/or paracrine manner as well as endocranially. Second, given the downregulation of trimeric adiponectin in the abdominal adipose tissue of the obese women who had delayed wound healing, the trimer form might act as an adipokine to locally regulate the function of adipose tissue, especially in promoting wound healing. Further studies with animal models should be carried out to determine if the trimer acts as an autocrine/paracrine factor to promote wound healing.

Since there were no significant differences in adiponectin at mRNA levels among the three groups studied, the reduction of trimer expression in the adipose tissue of obese women with delayed wound healing could be due to impaired post-translational modification and multimerization of adiponectin. Adiponectin multimerization is regulated by endoplasmic reticulum (ER) chaperones, such as Ero-1α and ERp44 [40,41], and by disulfide bond A oxidoreductase-like protein (DsbA-L) [42]. In addition, calcium can promote formation of the HMW form by binding with adiponectin [43]. As we detected no significant differences in these regulatory molecules in the abdominal adipose tissue (data not shown), we anticipate that other unidentified factors may have contributed to the downregulation of trimeric adiponectin in obese patients with delayed healing.

Adiponectin receptor 1 (AdipoR1) and adiponectin receptor 2 (AdipoR2) are two major types of receptors for adiponectin [44,45]. It has been reported that both receptors are expressed in adipose tissues, but AdipoR1 are more abundant than AdipoR2 [46]. Additionally, AdipoR1 has higher binding affinity with globular and trimer forms of adiponectin compared to AdipoR2 [45]. It is interesting to note that the effects of adiponectin on wound healing were mediated via AdipoR1/AdipoR2 receptors and the ERK signaling pathway in mice and in cells [24,29]. Together with our current finding that the trimeric form of adiponectin was significantly decreased in adipose tissue of obese women with delayed wound healing, the potential autocrine and/or paracrine role of trimer adiponectin in promoting wound healing may be via an AdipoR1-dependent mechanism in human adipose tissues. Further studies with animal models and trimer form of adiponectin are needed to demonstrate this possibility.

There are several limitations of this study. First, the specimens evaluated in this study are all from adult women, and the findings may not be representative for men and young women. For example, adiponectin levels are extremely high compared with other adipokines and hormones in the circulation [22]. Despite a wide and stable physiological range of adiponectin (1.9–17 µg/mL) [22], women have significantly higher levels of adiponectin compared with men [47]. In addition, adiponectin levels increase with age [17], and the trend of increase in women is independent of age in healthy adult populations [18,48]. Second, the sample size is relatively small due to a limit in patient availability. Future studies with larger populations would also allow assessment of whether differences in age, menopausal status, diabetes status, cancer history, and degree of abdominal fat might have confounded our analysis of the relationship between adiponectin and wound healing.

In conclusion, we identified trimeric adiponectin as a specific isoform that is reduced in the abdominal adipose tissues of obese women with delayed wound healing. This study uncovers a potential role of the trimeric form in the wound healing process in humans. Since no treatment is clinically available for helping obese patients who suffer from severely delayed wound healing, identification of factors contributing to such delays is critical for developing therapeutic targets to combat this complication.

## Figures and Tables

**Table 1:** Anthropometric characteristics of the cohort studied.

	Patient #	Gender	Age (Years)	BMI (kg/m^2^)	Glucose (mg/dL)	Healing Days
Non-Obese-	1	Female	29	23	66	35
Normal	2	Female	36	21.1	80	28
(n = 3)	3	Female	22	23.57	88	21
Average			29 ± 7	22 ± 2	78 ± 9	28 ± 7
	4	Female	63	43.5	190	28
Obese-	5	Female	63	36	68	35
Normal	6	Female	38	47.2	111	34
(n = 5)	7	Female	41	52.87	104	35
	8	Female	59	32.9	N/A	42
Average			53 ± 12	43 ± 8	118 ± 44.5	35 ± 5
	9	Female	52	33.82	134	90
Obese-	10	Female	65	38.69	93	139
Delay	11	Female	56	39.6	95	89
(n = 4)	12	Female	44	41.1	201	90
Average			54 ± 4	41 ± 2	131 ± 43.7	100 ± 26

Data are presented as mean ± SD.
